# Learning by creating teaching materials: Conceptual problems and potential solutions

**DOI:** 10.3389/fpsyg.2023.1095285

**Published:** 2023-02-16

**Authors:** Keiichi Kobayashi

**Affiliations:** Faculty of Education, Shizuoka University, Shizuoka, Japan

**Keywords:** learning by teaching, teaching materials, preparing to teach, intentionality, student learning

## 1. Introduction

For decades, educational research has demonstrated that students benefit academically from acting as a teacher, that is, tutoring other students face-to-face (e.g., Allen, 1976; Ehly et al., 1987), teaching a teachable agent (e.g., Chase et al., 2009), providing instructional explanations in a non-interactive manner (e.g., Fiorella and Mayer, 2013; Hoogerheide et al., 2014), and studying learning materials for teaching (e.g., Bargh and Schul, 1980; Nestojko et al., 2014). Recently, Ribosa and Duran (2022a,b) added an overlapping but distinct category to the list: *learning by creating teaching materials*. Teaching materials include a variety of student-generated artifacts, such as video lectures, test questions, instructional texts, and educational games. It is hypothesized that students can learn effectively through creating teaching materials for others without teaching the others afterward or using those materials for teaching. Nevertheless, the basically same mechanisms (e.g., generative processing, retrieval practice, social presence; Fiorella and Mayer, 2013; Koh et al., 2018; Lachner et al., 2022) are assumed to underlie the learning effects of teaching and creating teaching materials. Ribosa and Duran's (2022a,b) suggestion is worthy of serious consideration in that it may extend the scope of research on learning by teaching. Unfortunately, there is something unclear about what the authors mean by “creating teaching materials”, causing conceptual problems for their attempt. In this paper, I illustrate the conceptual problems using Ribosa and Duran (2022b) meta-analysis as an example and propose potential solutions.

## 2. Ribosa and Duran's meta-analysis: An illustrative case of conceptual problems

The meta-analytic review by Ribosa and Duran (2022b) included a total of 23 relevant studies (62 group comparisons) and indicated that an average effect size for the learning benefits of creating teaching materials vs. business-as-usual or alternative interventions (e.g., restudying, practicing retrieval, tutoring face-to-face) was *g* = 0.17 with a 95% confidence interval ranging from 0.04 to 0.31, rather small in magnitude but still statistically significantly greater than zero. However, this meta-analysis has two conceptual problems, and therefore, their findings must be interpreted with the greatest caution.

One conceptual problem lies in the assessment of eligibility. As one of the criteria for the inclusion of candidate studies in their meta-analysis, Ribosa and Duran (2022b) noted that each study had to include groups “creating teaching materials with the aim that a real or imaginary addressee autonomously learns from it” (p. 3). Of the studies judged to be eligible, some explicitly instructed one or more groups of students to create materials for teaching a fictitious student (e.g., Fiorella and Mayer, 2013, 2014; Hoogerheide et al., 2014; Jacob et al., 2020; Lachner et al., 2021). Given that providing students with instructions to teach produces the condition in which they engage in an activity with the intention of teaching, or helping others learn, it seems reasonable to judge that these studies satisfy the inclusion criterion, **though the fictitiousness of addresses may decrease their intention to teach** (Ribosa and Duran, 2022b). By contrast, other studies did not inform any groups of students, in advance of their creation of materials, that the materials would be utilized for others' learning or confirm whether they had the intention to teach while creating the materials (e.g., Chang et al., 2010; Erdmann and March, 2014; Hsu and Wang, 2018). Although some of the student groups created the materials for their peers or potential audiences, it is uncertain whether they did it with the intention of teaching. These studies would be ineligible if the inclusion criterion were applied stringently. The generous assessment of eligibility implies that Ribosa and Duran's (2022a,b) conceptualization of learning by creating teaching materials attaches little weight to students' intention behind their creation of materials.

Ribosa and Duran (2022b) may possibly justify their judgment on the eligibility of each study, arguing that regardless of creators' intentions, student-generated materials, if publicized or shared in the class, can serve as educational resources from which someone learns autonomously. But still, in the context of learning by acting as a teacher, the intentionality of teaching should not be trivialized. Indeed, there is some evidence that students' intention to teach, including their expectation of teaching, is a determinant of their learning by teaching or preparing to teach. For example, in a study by Chase et al. (2009), students read a text and created a concept map in a computerized learning environment. The concept map represented knowledge in a computer character's brain and determined the character's performance in a subsequent gaming situation. Those who believed that they were teaching the character engaged more enthusiastically in the learning activity and learned better than those who believed that they were working for their avatars. Similar results were reported by Silvervarg et al. (2021). Furthermore, several studies have shown that expecting to create an instructional video for a fictitious student increases one's preparatory learning more than expecting to take a test (Fiorella and Mayer, 2013, 2014; Guerrero and Wiley, 2021).

Another conceptual problem concerns how the learning effects of creating teaching materials were estimated in the meta-analysis. Ribosa and Duran (2022b) adopted the following group comparisons as samples: “As for intervention groups,... this meta-analysis considered any group that created a teaching material—with or without preparing-to-teach. As for control groups, this meta-analysis considered all the groups that did not involve the creation of a teaching material” (p. 11). In this context, *preparing-to-teach* refers to studying with the expectation of teaching. Accordingly, as shown in Table 1, the meta-analysis included but did not differentiate among four types of group comparison that are defined by 2 (intervention group: creating teaching materials with or without studying beforehand for teaching) × 2 (control group: studying for teaching or not without creating teaching materials afterwards). This mixing is problematic, considering that, at least under some conditions, students learn effectively through merely studying for teaching (e.g., Bargh and Schul, 1980; Benware and Deci, 1984; Nestojko et al., 2014; Muis et al., 2016; for a meta-analytic review, see Kobayashi, 2019). The learning effects estimated from the four types of group comparison may be conceptually heterogenous.

**Table 1:** Four types of group comparison included in Ribosa and Duran (2022b) meta-analysis.

	**Intervention group**		**Control group**		
**Types**	**Studying for teaching**	**Creating teaching materials**	**Studying for teaching**	**Creating teaching materials**	**Examples**
A	Included	Included	Included	Not included	id. 23 (Hoogerheide et al., 2014; 1)
B	Included	Included	Not included	Not included	id. 36 (Jacob et al., 2020)
C	Not included	Included	Included	Not included	id. 12 (Fiorella and Mayer, 2014; 2)
D	Not included	Included	Not included	Not included	id. 53 (Lachner et al., 2021; 1)

Each example comes from Table 3 in Ribosa and Duran (2022b).

More importantly, Ribosa and Duran's (2022b) meta-analysis was based on the assumption that the products of studying for teaching are excluded from the creation of teaching materials. Although the authors noted that control groups “did not involve the creation of a teaching material” (p. 11), groups of students who studied for teaching were compared with intervention groups (types A and C in Table 1), suggesting that the students were not considered to create teaching materials. Still, even while studying learning materials, students with the intention of teaching can set about creating teaching materials. For example, they may select important information from the learning materials and organize the selected information from a teacher's perspective (Nestojko et al., 2014), thereby getting ready for explaining on video (Fiorella and Mayer, 2013; Muis et al., 2016). If so, studying for teaching entails the partial and covert creation of teaching materials. This means that in type A and C group comparisons, the entire process of creating materials with the intention of teaching (intervention groups) was compared with the partial process (control groups). The two types of group comparison are inappropriate for the estimation of the effectiveness of learning by creating teaching materials (vs. business-as-usual or alternative interventions), provided that engaging in the creation process, including preparing to teach, produces the learning effects.

## 3. Discussion

Based on the critical examination of Ribosa and Duran's (2022b) meta-analysis, I propose that the intentionality of teaching, as well as student-generated materials, is the key to the conceptualization of learning by creating teaching materials. More specifically, students' creation of teaching materials is distinguishable by their intention to teach rather than by the possibility of serving for others' autonomous learning. Student-generated materials are categorized as those for teaching only when students intend to teach others by providing the materials directly or indirectly. Moreover, students' acts of creating teaching materials begin with their intention to teach, for example, when they study learning materials with teaching expectancy and when they are told to create the teaching materials (unless the opportunity of studying for teaching is given in advance). The process of learning by creating teaching materials includes preparing for teaching but not studying beforehand without the intention of teaching.

Emphasizing the intentionality of teaching resonates with the methodology of research on learning by teaching, in which students who fill the role of teacher in a learning-by-teaching activity are instructed to study for teaching and/or to teach others (see e.g., Bargh and Schul, 1980; Ehly et al., 1987; Chase et al., 2009; Veloso et al., 2019). This methodology presupposes that teaching is a goal-directed and intentional process. As noted previously, whether students engage in a learning activity with or without the intention of teaching has been shown to be a crucial factor in their learning. Developmental research on teaching has also found that even preschoolers can adapt their communication to pedagogical contexts if they are told to teach (Gelman et al., 2013; Calero et al., 2015; Rhodes et al., 2015). For example, Baer and Friedman (2018) had 4- and 5-year-olds teach or tell their knowledge about an object (e.g., an umbrella with a frog appearance) to others. The teaching group included generalizable information [e.g., “umbrellas keep you dry” (p. 465)] in their explanations more frequently than did the telling group. No significant difference was found between the two groups in the inclusion of specific information [e.g., “the umbrella looks like a frog” (p. 465)]. These findings suggest that one's intention to teach acts as a trigger for the processes characteristic of teaching (e.g., communicating generic or generalizable knowledge to less knowledgeable others). To analyze “student creation of teaching materials through the lens of learning by teaching” (Ribosa and Duran, 2022b, p. 2), researchers should differentiate clearly between learning by creating materials with and without the intention of teaching.

Additionally, the proposed conceptualization of learning by creating teaching materials assumes the potential contribution of preparing to teach. In line with this assumption, evidence suggests that the learning effects of creating teaching materials differ depending on whether and how students study beforehand for teaching. For example, Fiorella and Mayer (2014) found that learning by explaining on video worked effectively after studying with teaching expectancy but not after studying with test expectancy. In a study by Kobayashi (2021), students who had studied beforehand for teaching, in collaboration with their partners, learned better through recording instructional explanations on video than those who had studied individually. Of course, when teaching materials are created, preparing to teach is not limited to studying beforehand for teaching. In some cases, students may undertake preparation (e.g., brainstorming, planning, replanning) in parallel with the other processes of creating teaching materials, such as recording, writing, drawing, and programming (e.g., Slussareff and Boháčková, 2016). Currently, we have no data regarding whether and how preparing to teach affects one's learning in such cases. Having said that, in research on learning by creating teaching materials, it is students with limited domain knowledge and experience in teaching who act as teachers (Fiorella and Mayer, 2013, 2014; Hoogerheide et al., 2014). Students' creation of teaching materials requires that they acquire some knowledge about the subject matter and transform it into to-be-taught information, regardless of whether they have an opportunity to study beforehand for teaching. There is no a priori reason to disregard the role of preparing to teach in learning by creating teaching materials.

In conclusion, I agree with Ribosa and Duran (2022a,b) that activities in which students create materials for teaching afford them an opportunity to learn effectively. However, as illustrated in the second section, the authors are unclear about the nature and extent of students' creation of teaching materials, thereby introducing confusion into their studies and probably future research. My proposal that the process of creating teaching materials is defined by one's intention to teach and includes preparing to teach will contribute to solving the conceptual trouble and advance our understanding of learning by creating teaching materials.

## Author contributions

KK designed the paper, analyzed the literature, and drafted the manuscript.
